# Thermal Deformation in Non-Planar Large-Scale Additive Manufacturing of ABS: Experimental and Finite Element Analysis

**DOI:** 10.3390/ma19061064

**Published:** 2026-03-11

**Authors:** Mehmet Aladag, Engin Tek, Mehmet Ali Akeloglu, Adrian Dubicki, Izabela Zgłobicka, Omer Eyercioglu, Krzysztof J. Kurzydlowski

**Affiliations:** 1 Faculty of Mechanical Engineering, Bialystok University of Technology, Wiejska 45C, 15-351 Bialystok, Poland; a.dubicki@pb.edu.pl (A.D.); i.zglobicka@pb.edu.pl (I.Z.); k.kurzydlowski@pb.edu.pl (K.J.K.); 2 Department of Electronics and Automation, Gedik Vocational School, Istanbul Gedik University, 34876 Istanbul, Türkiye; engin.tek@gedik.edu.tr; 3 Mechanical Engineering Department, Engineering Faculty, Gaziantep University, 27410 Gaziantep, Türkiye; makeloglu@gantep.edu.tr (M.A.A.); eyercioglu@gantep.edu.tr (O.E.)

**Keywords:** hybrid large-scale additive manufacturing, non-planar printing, coating, numerical simulation, thermal deformation

## Abstract

In this study, thermal deformation in non-planar, large-scale additive manufacturing (LSAM) was experimentally and numerically investigated. A Bézier-based non-planar build surface was fabricated by CNC machining, and a single layer of ABS was deposited using a hybrid LSAM system. Toolpaths with raster angles of 0° and 45° were generated for surface-conformal printing. Infrared thermography was employed to monitor the thermal history during deposition. A three-dimensional finite element model was developed to simulate transient heat transfer and thermally induced deformation. Experimental deformation was quantified by 3D scanning and compared with simulation results. The results show that the slope geometry strongly influences deformation direction: negative slopes promote contraction, whereas positive slopes lead to upward deflection. Maintaining the material temperature above the glass transition temperature significantly reduces skew deformation. The finite element method predictions demonstrate strong agreement with experimental measurements, with normalized root mean square errors (NRMSEs) of approximately 11% for thermal deformation and 10% for temperature history. The proposed framework enables prediction and mitigation of thermal warping in non-planar polymer additive manufacturing.

## 1. Introduction

Additive manufacturing (AM) by the material deposition method is one of the suitable methods for manufacturing polymer parts. As such, expectations for 3D printers are evolving on a daily basis. Because the industry expects to produce large parts in one piece (without assembly) with 3D printers, the solution lies in adapting the size of the printers and the printing parameters. Moreover, the parts produced by the large-scale additive manufacturing method are subjected to a finishing process for end-use. The large diameter of the extruder nozzle makes the layers visible. Additionally, on inclined or non-planar surfaces produced via planar slicing, a stair-step artifact becomes more prominent as the surface slope relative to the deposition plane decreases. This physical phenomenon is a result of the discrete layer-by-layer approximation of curved geometries, which negatively affects surface roughness and mechanical integrity of parts [1].

Even though planar printing is the most ideal and is commonly used nowadays, it also has its cons. One of the drawbacks is the esthetic appearance of the sloped or curved surfaces, which can be attributed to the stair-stepping effect. The planar printing affects the surface roughness negatively, and it also affects the mechanical properties [2]. Thus, planar printing is not satisfactory in terms of esthetic appearance and mechanical properties in some cases. Researchers have explored methods to mitigate the stair-step effect through post-processing operations, including milling, grinding, and painting. However, these approaches often result in changes to the model’s geometry. However, this approach has not become satisfactory. To address this issue, a new slicing method called non-planar 3D printing has been proposed to improve the printing quality. Thus, a tunable slicing method was presented by researchers; non-planar 3D printing has been proposed to improve the printing quality [3,4,5]. Several studies have been carried out on non-planar 3D printing till now [6,7,8,9,10,11]. Before these studies, non-planar printing was first proposed by Chakraborty [12]. Following this, Ahlers et al. [6] developed an algorithm for open-source slicing software (Slic3r v1.2.9-stable). In their algorithm, besides non-planar surface toolpath generation, an adaptive slicing algorithm was developed. With this algorithm, printing time was decreased, and geometric esthetic appearance was enhanced [6,13].

In general, in conventional additive manufacturing, the extruder nozzle axis remains perpendicular to the X–Y plane (the building bed). Consequently, the nozzle orientation is fixed along the *Z*-axis, which limits the trajectory when complex parts have undercut geometries. The trajectory of the extruder is limited when the complex parts have an undercut geometry [14,15,16]. The slicer software generates support to build these undercut geometries, particularly for the building of overhanging structures [17,18]. These supports result in additional fabrication time and material waste, increasing costs. Optimized support design can, however, prevent time and material waste [19,20]. Despite these advantages, material-specific challenges persist [21]. Thermally induced defects and warping remain the primary limitations in the fabrication of large-scale polymer components [22]. Due to the positive thermal expansion of most polymers, the appropriate cooling process plays a significant role.

Recent investigations have been mentioned regarding thermal defects in printing. The key point is the cooling time and ambient temperature in printing [23,24]. Compton et al. [25] studied the thin-wall thermal evolution of the CF-ABS mixture produced in large-scale AM using infrared imaging. In this regard, a one-dimensional transient thermal model was developed. The model predictions are in excellent agreement with the observed temperature profiles. Based on these results, we have developed criteria to guide the selection of deposition parameters that minimize the possibility of cracking during printing. Another researcher focused on the additive material effects on the thermal warpage [26,27,28,29,30]. Kim et al. [31] developed a thermomechanical model for large-scale additive manufacturing to compare the results with an experimental investigation of the composition of carbon fiber, utilizing a de-homogenization process with a 20% ABS weight. The carbon fiber reduced distortions. These defects can be simulated by the analytical method and the finite element method. Regarding this, the failure models of a thermal simulation were made by Akbari et al. [32]. In this study, the researcher focused on the FEA parameters of the thermal analysis. The simulation and experimental results agreed with each other when considering the proposed simulation criteria [32]. Polyzos et al. [33] investigated nylon reinforced with continuous carbon fiber. They developed a mathematical model and compared it to the experimental results. The simulation results were consistent with the experimental findings. The simulation results revealed the temperature and distortion patterns from the experiment.

Manufacturing large-sized parts requires more effort than small-sized parts, with a large material deposition rate in the AM method [1,34]. This is because production is conducted with unspecified parameters. In other words, numerous studies have been conducted on 3D printers to date, but the parameters obtained in these studies have been primarily adapted for small-scale 3D printers [35,36,37,38,39]. There is no suitable software that slices the model in order to generate a toolpath while controlling the parameters for LSAM. Even if there is software, existing LSAM systems typically utilize their proprietary commercial software. At this stage, researchers strive to determine suitable printing parameters for LSAM systems.

Although thermal deformation has been extensively studied for planar FDM and large-scale printing, the thermal behavior of surface-conformal non-planar printing remains largely unexplored. In particular, the influence of local surface slope on deformation direction and magnitude has not been experimentally quantified. Furthermore, validated numerical models for predicting thermal warping in non-planar LSAM are scarce. This study addresses these gaps by combining infrared thermography, 3D scanning, and finite element modeling to analyze thermal deformation in non-planar surface-conformal printing of ABS.

In the present study, non-planar surface-conformal 3D printing was performed, where the preliminary surface geometry was generated based on a Bézier curve model. As a result, the surface included both positive and negative slopes, introducing varying degrees of complexity for the additive manufacturing process. A single layer of acrylonitrile butadiene styrene (ABS) was deposited on these non-planar surfaces at different raster angles (0–90°), enabling a detailed investigation of how raster orientation affects material behavior during printing. The influence of thermal deformation due to surface angle and raster angle was assessed using thermal imaging captured during the printing process. This allowed for real-time monitoring of heat distribution and deformation tendencies. Additionally, a finite element model (FEM) was developed to simulate the thermal response and predict deformation. The thermal analysis results from the FEM were then compared with experimental data, offering a comprehensive evaluation of the interaction between material deposition, surface geometry, and thermal deformation. This approach will provide a robust understanding of the deformation mechanisms in non-planar additive manufacturing, and it will offer to optimize the parts’ hybrid manufacturing processes.

## 2. Materials and Methods

### 2.1. Printing Material

The material of the sample is acrylonitrile butadiene styrene (ABS) thermoplastic polymer, with the properties shown in Table 1. The granular form of material was dried at 80 °C for 4 h in an industrial oven before use to decrease the humidity. Then, the material was deposited at 240 °C onto a non-planar wooden surface.

### 2.2. Hybrid-LSAM System

**Figure 1:**
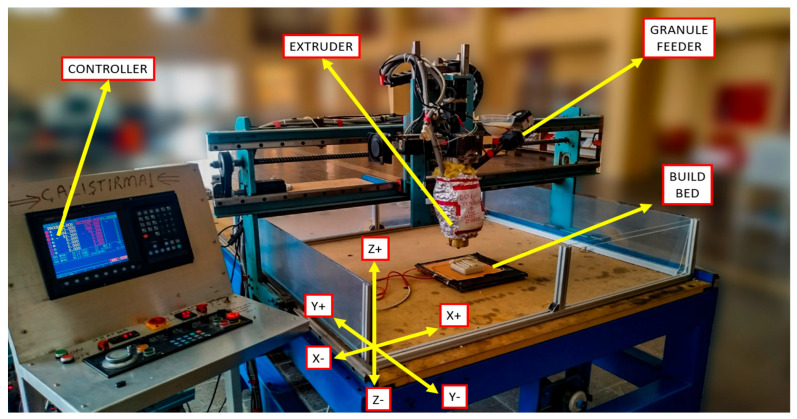
LSAM system in the mechanical engineering laboratory, Gaziantep University [1].

The Hybrid-LSAM system is essentially a 3-axis CNC router that has been modified to enable the printing of non-planar objects. Figure 1 shows the LSAM system, which has been designed and manufactured to replace the spindle of the 3-axis CNC unit. The maximum displacements in the X, Y, and Z directions are 1800, 2500, and 400 mm, respectively. All movement axes are driven by a high-resolution servo system, which enables the simultaneous operation of all servo motors and allows for printing on non-planar surfaces.

The extruder used in the system is a single-screw extruder that is powered by a variable-speed motor. ABS granules are fed into the extruder through an automatic feeder. The feed rate of the granules and the speed of the screw can be controlled to ensure that molten polymer is deposited at a rate consistent with the movement of the axes and the desired bead profile.

The barrel of the extruder is equipped with band heaters and a control unit to maintain the required temperature range for the chamber and nozzle. Additionally, to maintain a consistent ambient temperature, the printing area was covered, and a heated bed was placed inside the printing system. Thus, the environmental temperature was kept at a constant level. Therefore, the amount of deflection caused by fast cooling was decreased.

### 2.3. Non-Planar Build Plate

**Figure 2:**
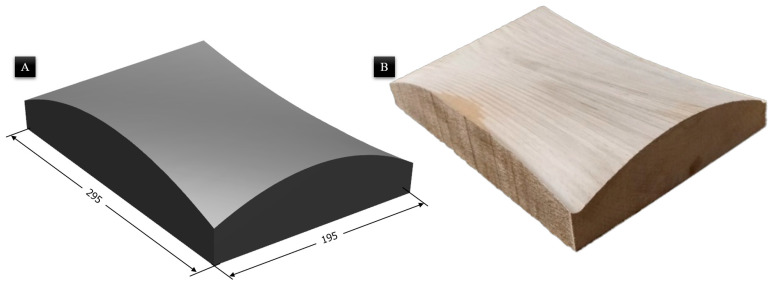
Non-planar build bed. (**A**) CAD view with dimensions in mm and (**B**) bed manufactured from wood.

A parametric Bézier surface geometry was used as a non-planar, build-plate surface. After that, the part was built on this geometry. This is also referred to as surface-conformal printing in the literature. Wood was selected as the substrate material due to its ease of machining into complex Bézier geometries and its low thermal conductivity, which acts as a thermal insulator. While wood presents challenges in achieving rapid, uniform bed temperatures, its insulating nature reduces the initial heat loss from the deposited ABS bead to the substrate. This assists in maintaining the material temperature above the glass transition during the critical initial phases of deposition. The size of the non-planar build plate is 300 mm in the X direction and 200 mm in the Y direction, respectively. The build plate image is given in Figure 2.

### 2.4. Non-Planar Toolpath Generation

**Figure 3:**
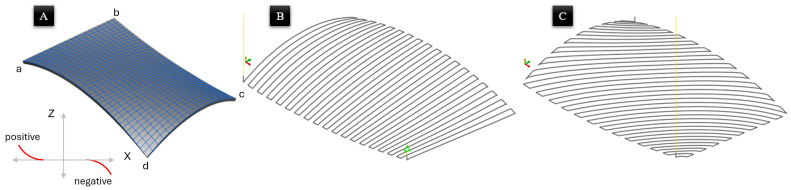
(**A**) Bézier surface CAD model, (**B**) toolpath for raster angle 90, and (**C**) toolpath for raster angle of 45. Slopes at corners a, c, and d are negative (declining), and corner b is positive (inclining) relative to the toolpath start.

As the objective of the experiment was to execute a surface-conformal 3D printing process, a single layer was produced. The Bézier surface was designed using SolidWorks 2020 Service Pack 2 software and was exported in the “Standard Triangle Language” (STL) file format. The next step involved transferring the model to the Slic3r v1.2.9-stable slicer software to generate the non-planar toolpath (as shown in Figure 3). Subsequently, various printing parameters, including nozzle diameter, layer thickness, printing temperature, and printing speed, were defined. In addition, the non-planar slicing parameters were determined based on the LSAM machine system. The values and parameters were tabulated in Table 2.

To facilitate the analysis of thermal deformation, the local surface slopes are defined relative to the nozzle’s travel vector. A positive slope represents an increase in height (Z) along the printing direction, whereas a negative slope represents a decrease in height. Based on this convention, the surface gradients at corners “a”, “c”, and “d” are characterized as negative, while corner “b” represents a positive slope. This directional definition is critical for interpreting the resulting contraction and upward deflection trends observed in the experimental samples.

### 2.5. FEM Simulation Model

A three-dimensional transient thermomechanical finite element model was developed using MSC Marc/Mentat v2021.4 software to simulate the thermal evolution and resulting deformation of a single non-planar deposited layer. The deposited ABS layer and the wooden build plate were modeled as deformable bodies with temperature-dependent thermal behavior. The wooden build plate was modeled as a linearly elastic body with isotropic thermal properties. Although wood is naturally orthotropic, an isotropic assumption was adopted as a suitable simplification. This is justified because the thermal expansion of the ABS material is the primary driver of deformation in this study. Furthermore, the substrate maintains a relatively stable thermal state compared to the transient cooling of the deposited layer, making the impact of orthotropic substrate variations negligible for the predicted global deformation trends.

The printed layer geometry was numerically reconstructed based on the parametric Bézier surface used in the experimental phase. To ensure computational efficiency and result accuracy, a structured quadrilateral mesh was utilized to discretize the deposited layer. Simultaneously, the wooden build plate was discretized using hexahedral elements. Based on a preliminary mesh convergence analysis conducted to ensure numerical stability, the final model consisted of approximately 35,000 elements with an average element size of less than 2 mm.

**Figure 4:**
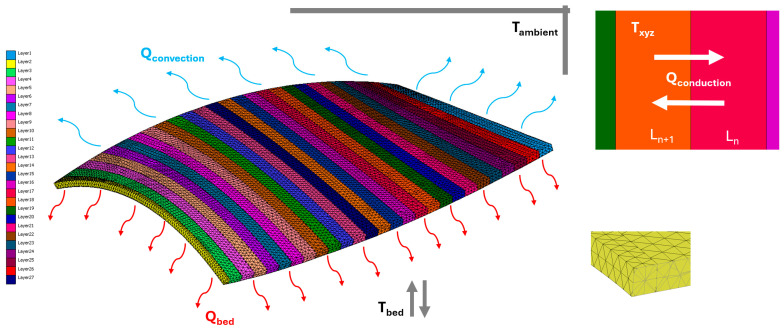
Mesh and boundary conditions of the model.

To accurately simulate the progressive material deposition, the single physical layer was discretized into 27 individual bead segments. These segments were activated sequentially along the toolpath across 27 load cases, each representing 77.7 s, to replicate the 35 min experimental duration. Within each finite element, the thermal state was governed by a coupled mechanism of internal conduction and surface-to-ambient convection and radiation. Heat input was modeled using a time-dependent thermal volume flux of 0.21 W/mm^3^, which was applied as a pulse for the first 2 s of each segment’s activation to mimic the nozzle’s transit before allowing natural cooling. The comprehensive mesh morphology and assigned thermal boundary conditions are illustrated in Figure 4.

To model the thermal behavior of the printing process in a 3D Cartesian coordinate system (x, y, and z), where ABS material is being printed on a general wooden build plate, the heat conduction equation can be employed to describe the heat flow within the system. The 3D heat conduction equation is given in Equation (1). Newton’s law of cooling described the convection process (refer to Equation (2)), while Fourier’s law governed the conduction process (refer to Equation (1)).

(1) $$\rho c\frac{\partial T}{\partial t}=\nabla \cdot \left(k\nabla T\right)+Q$$

where *ρ* is the material density, *c* is the specific heat capacity, *T* is the temperature, *t* is time, *k* is the thermal conductivity, and *Q* is any heat source/sink term.

At the initial condition, the thermal boundary is $T_{x,y,z,0}=T_{initial}$, where *T_initial_* is the initial temperature distribution. The *x* and *y* present the direction components of heat transfer in 3D Cartesian coordinates. The boundary condition in the bed, $T_{x,y,t}=T_{bed}$, was defined. The building bed temperature was nominated to *T_bed_*. The boundary condition of the printing temperature is presented as $T_{x,y,z,t}=T_{printing}$ where *T_printing_* is the nozzle temperature.

To account for temperature variations, specific temperature loads were defined. During printing, the deposition temperature was set to 240 °C, while the ambient temperature was maintained at 30 °C. To maintain thermal stability, the thermal boundaries of the layer were set up. Conductive boundaries were established at the bottom, connecting the layer to the building bed, while convective boundaries interacted with the air at the top of the layer.

Upon completion of the printing process, a cooling mechanism was employed. By applying the cooling process, the temperature of the layer was gradually brought down to room temperature, ensuring a controlled and stable thermal outcome. This process is expressed with Equation (2):

(2) $$Q_{conv}=h\cdot A\cdot ∆T$$

where $Q_{conv}$ is the convective heat transfer rate, *h* is the convective heat transfer coefficient, and *A* is the surface area through which heat is transferred, ∆T is the temperature difference between the surface and the surrounding air (refer to Equation (3)).

(3) $$Q_{cond}=-k\cdot A\cdot \frac{dT}{d_{x,y,z}}$$

where *Q* is the conductive heat transfer rate, *k* is the thermal conductivity of the material, and *A* is the cross-sectional area through which heat is conducted, and the $\frac{dT}{d_{x,y,z}}$ temperature gradient in the material is the temperature difference along the direction of heat transfer.

The thermal evolution of the part is governed by the transient interaction between internal conduction and surface convection. Within the deposited bead, heat is redistributed according to the material’s thermal conductivity, while heat is simultaneously dissipated to the environment via Newton’s law of cooling. To evaluate the temperature distribution within the 4 mm thick layer, the Biot number (Bi) was considered. Due to the low thermal conductivity of ABS (0.177 W/mK) and the large characteristic length of the LSAM bead, Bi indicates that significant internal temperature gradients exist. Consequently, a 3D transient conduction model was necessary to capture the vertical and transverse thermal gradients that drive deformation.

Moreover, precise thermal definitions were assigned to each element composing the entire layer. Specifically, boundary conditions for all layers, encompassing both conduction and convection, were applied. The simulation was executed by systematically activating element groups sequentially along the toolpath. Surface convection boundary conditions were dynamically applied to the exposed top and side faces of each segment immediately upon its activation. This ensured that the model captured the transient heat loss occurring during the deposition process itself. Once the final segment was activated and the toolpath was completed, the cooling phase was initiated, allowing the entire geometry to reach ambient temperature.

### 2.6. Thermal Deformation Measurement

The geometrical evaluation of the printed part was conducted by comparing the manufactured sample and FEA results to the original CAD model. Accurate measurement of geometry is particularly challenging for complex structures when using traditional methods. Therefore, the printed samples were scanned using an Artec Eva (Artec Europe S.à r.l., Senningerberg, Luxembourg) 3D scanner, and the acquired data were converted into STL surface models. Subsequently, using SolidWorks 2020 Service Pack 2 CAD software, the printed sample, the as-designed CAD model, and FEA results were aligned and fitted to one another.

The alignment between the 3D-scanned STL models, the FEA results, and the original CAD model was performed using a Global Best-Fit algorithm within the CAD software. To ensure accuracy, a three-point alignment was first executed using the fixed corners of the wooden substrate as reference datums, followed by an iterative optimization to minimize the global deviation. This dual-step process ensured that the resulting surface deviation maps accurately represent the thermally induced warping relative to the intended design.

To quantitatively assess the agreement between FEA predictions and experimental measurements, the deformation values were extracted along four edges (Front, Rear, Left, and Right) at uniform intervals of 20 mm. The predictive accuracy of the FE model was evaluated using several statistical metrics: root mean square error (RMSE), mean absolute error (MAE), and the Pearson correlation coefficient (r). Additionally, the normalized root mean square error (NRMSE) was calculated as the ratio of RMSE to the total experimental deformation range (D_max_ − D_min_), providing a scale-independent measure of prediction accuracy.

## 3. Results and Discussion

### 3.1. Printed Samples

**Figure 5:**
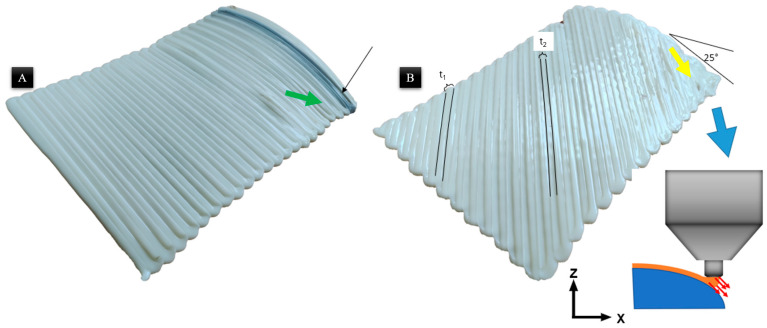
Printed samples: (**A**) raster angle 90°, (**B**) raster angle 45°.

Non-planar, surface-conformal printing was carried out for two raster angles. The images of printed samples are shown in Figure 5. The current analysis investigates the influence of transverse surface gradients on thermal deformation. Upon experimental investigation, the observed phenomena revealed complexities beyond the initial theoretical framework. The effect of the slope is obviously important in terms of the material rheology and the instantaneous orientation of the nozzle. This is a limitation of the three-axis 3D printing system, and it was discussed widely in the previous article related to this study [1].

The encountered defects in the printing with Hybrid-LSAM were marked in Figure 5. Both printed samples have common defects; however, some unique defects were observed in the sample with a raster angle of 45°. The surface with a higher slope (25°) was observed to have material swept due to the nozzle orientation.

The physical defects observed in the Hybrid-LSAM samples are primarily attributed to the kinematic limitations of the three-axis system on non-planar geometries. While the overlap distance remained constant, the deposited beads exhibited asymmetric superimposition on the 25° sloped regions. Because the nozzle orientation remains fixed along the *Z*-axis, it is not normal to the curved surface; this creates a variable standoff distance across the bead width (Figure 5B). Moreover, the width of the bed in the middle of the part (t_2_) is lower than in the beads with a high slope angle (t_1_). Consequently, the molten ABS tends to flow toward the direction of gravity, leading to non-uniform layer thicknesses and a reduction in bead width compared to the planar regions (Figure 5B indicated with a yellow arrow). This effect is most pronounced at the 45° raster angle, where the toolpath vector compounds with the surface gradient, leading to significant material “sweeping”.

### 3.2. Geometrical Inspection

The thermal deformation results, predictions using FEA, and those on printed samples were compared with the as-drawn CAD model. The amount of deformation was visualized and is given in Figure 5. In order to discuss results efficiently, the corner slopes of the geometry were defined as A, C, and D were negative, and B was a positive angle.

**Figure 6:**
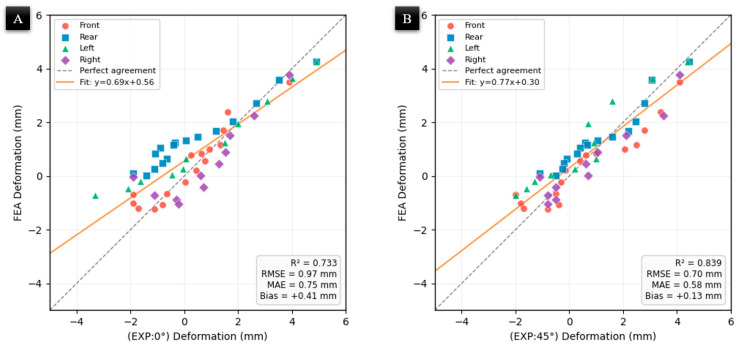
Correlation between FEA-predicted and experimentally measured thermal deformation for all four edges: (**A**) FEA vs. raster angle 0°, (**B**) FEA vs. raster angle 45°.

The FEM predictions and experimental results exhibited strong correlation (Figure 6), with Pearson correlation coefficients of r = 0.89 and r = 0.92 for raster angles 0° and 45°, respectively (R^2^ = 0.73 and 0.84). The root mean square error (RMSE) between FEA and experimental deformation was 0.97 mm for raster angle 0° and 0.70 mm for raster angle 45°, corresponding to a normalized RMSE (NRMSE) of approximately 11%, calculated as RMSE divided by the total experimental deformation range (D_max_ − D_min_). The FEA model also exhibited a slight systematic positive bias of 0.41 mm and 0.13 mm for raster angles 0° and 45°, respectively, indicating that the simulation marginally overestimates deformation magnitude. This bias is attributed to idealized boundary conditions in the FE model, which do not fully capture the kinematic limitations of the three-axis system. Specifically, the system’s inability to maintain a normal nozzle orientation on steep slopes leads to material “sweeping”, creating physical discrepancies in layer thickness that exceed the resolution of the structured quadrilateral mesh used in the simulation.

As demonstrated in Table 3, the proposed MSC Marc/Mentat framework achieves an 11% error margin. This places our model’s accuracy squarely in the upper tier of current state-of-the-art methodologies—highly comparable to specialized Abaqus and Digimat simulations, which report errors in the range of <1% to 12%, and demonstrating greater stability than the wide variance (2.5% to 60.9%) observed in some ANSYS-based approaches. Overall, this confirms that the proposed framework provides a reliable methodology for predicting complex thermally induced warping in non-planar large-scale additive manufacturing.

**Figure 7:**
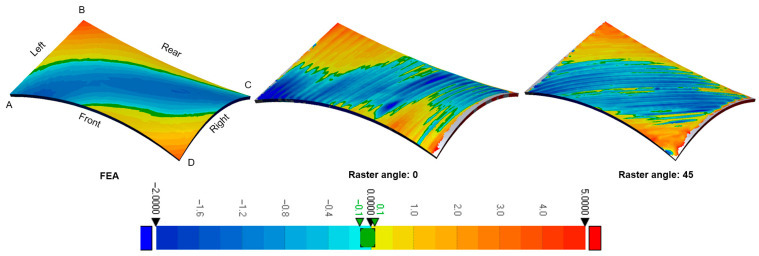
Surface deviation map comparing FEA predictions with experimental samples (0° and 45° raster angles). The color scale represents geometric deviation in mm from the nominal CAD surface.

The positive and negative slopes and their antipodal deformations were in the same direction. The deformation tendency was seen toward downward in the negative-slope surface (Figure 7, corners A–C), and vice versa in a positive-slope surface as well (Figure 7, corner B). The observed upward deformation on positive slopes is attributed to non-uniform cooling across the layer thickness, which induces a bending moment due to thermal gradients combined with gravitational flow of the molten polymer. Interestingly, there is an exception in that, while the corner D had a negative slope, the deformation direction was upward. One reason can be suggested for this exception; the deflection along the corners A to C was negative. The reason for this could be folding due to the time-dependent cooling; hence, it might be caused by antipodean-angled anisotropic folding.

**Figure 8:**
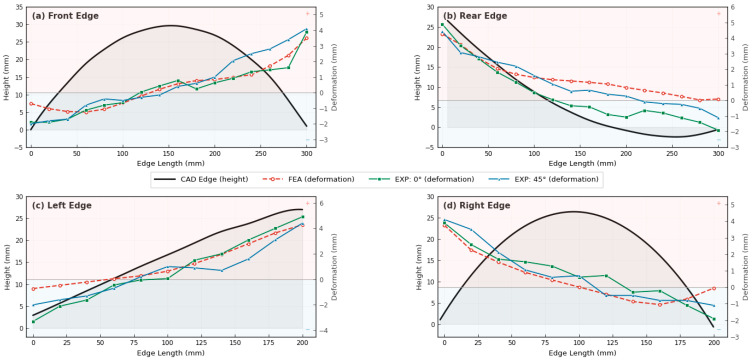
Dual-axis comparison of edge profile geometry and thermal deformation along four edges: (**a**) Front, (**b**) Rear, (**c**) Left, and (**d**) Right. The left *y*-axis represents the as-designed CAD edge height (black solid line), and the right *y*-axis represents the thermal deformation measured from FEA (dashed red), experimental raster angle 0° (green), and experimental raster angle 45° (blue). The shaded regions indicate positive (upward) and negative (downward) deformation directions.

The amount of thermal deflection of both raster angles, 0° and 45°, in the printed samples does not differ significantly. However, when comparing the color distribution of the amount of deflection, the negative deflections were observed to be wider. Another finding was the similarities in the entire antipodean deflection value of the edges. The plots in Figure 8 show this thesis more clearly. Four edges of the mode were plotted in this figure. In the plot, the left-hand axis shows absolute height, and the right-hand axis shows the deformation. The black solid line presents the as-designed CAD model. The red dashed line shows the FEA results, the green dashed line shows the printed sample with raster angle 0, and the other one shows the printed sample with raster angle 45. The positive deflection values were up to 4 mm, and the lower deviation values were up to 2 mm. In other words, the maximum and minimum deviations of 4 mm and 2 mm were essentially seen in all edges.

The maximum thermal deformation was observed at the corners. The reason is the maximum surface area. In other words, the two corner edges are open; therefore, the deposited material experiences maximum heat loss. Accordingly, fast cooling led to faster thermal contraction.

### 3.3. Thermal Imaging

**Figure 9:**
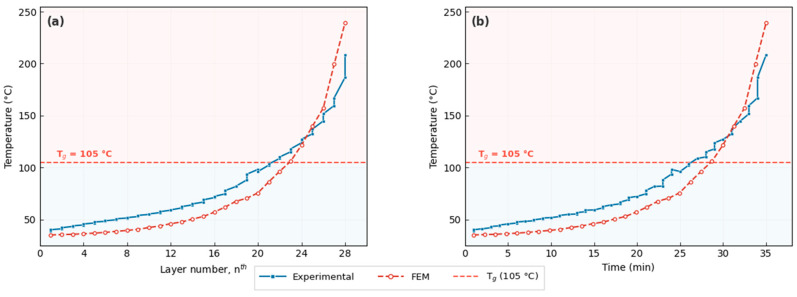
Thermal history during non-planar deposition comparing experimental (infrared thermography) and FEM simulation results: (**a**) temperature evolution as a function of layer number and (**b**) temperature evolution as a function of time. The dashed red line indicates the glass transition temperature (T_g_ = 105 °C) of ABS.

The thermal solidification history is critical to understanding the resulting deformation. Figure 9 illustrates the transient temperature profile of the material during the 35 min deposition process, comparing both experimental measurements from infrared thermography and FEM simulation predictions. As the printing progresses, the accumulated heat from subsequent beads maintains the temperature of the previously deposited material. According to the data in Figure 9a, the experimentally measured local temperature crosses the glass transition threshold at approximately the 20th layer segment (roughly 25 min into the process, Figure 9b). The FEM simulation captures the same overall trend; however, it predicts a lower thermal accumulation in the early-to-mid layers, with the T_g_ threshold crossed at approximately the 23rd layer. This discrepancy is attributed to the fact that the infrared camera captures the surface-averaged temperature over a finite area, whereas the FEM reports the nodal temperature at a discrete point, and the idealized convection boundary conditions in the simulation may overestimate the heat loss in the early stages of deposition. Despite this offset, both experimental and numerical results converge toward similar peak temperatures in the final layers, confirming that the FEM reliably captures the critical thermal regime governing deformation. This prolonged period above T_g_ is beneficial as it allows for stress relaxation, which significantly reduces the final warping magnitude in those regions.

**Figure 10:**
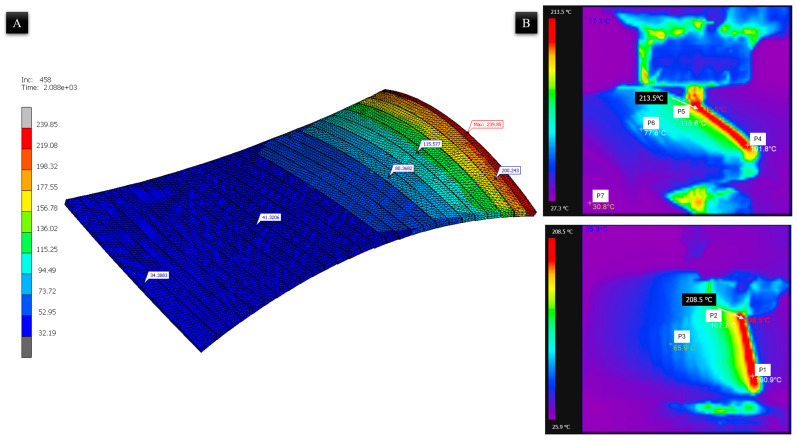
Temperature distribution during non-planar deposition: (**A**) FEM simulation showing the thermal field, and (**B**) infrared thermography captures at two nozzle positions.

The thermal deformation of the printed part is fundamentally dependent on the thermal solidification history of the deposited material. Real-time monitoring via infrared thermography reveals significant thermal interactions between the nozzle and the previously deposited beads (Figure 10). As shown in the thermal images, the area immediately behind the current nozzle position remains at an elevated temperature. Specifically, points P2 (107.7 °C) and P5 (115.6 °C) are observed to be above the T_g_ of 105 °C while the nozzle is at positions P1 and P4, respectively. The FEM temperature distribution (Figure 10A) corroborates this observation, showing a comparable thermal gradient along the deposition path, with temperatures ranging from 34.4 °C in the earliest deposited region to 239.9 °C at the active deposition front. This confirms that the currently deposited molten material transfers heat back into the preceding segments, acting as a secondary heat source that delays the cooling process.

The maintenance of the material above its T_g_ for this extended duration is critical for minimizing thermal defects. It allows for the relaxation of thermally induced stresses while the ABS remains in a rubbery state before transitioning into a rigid, glassy state. The FEM thermal history showed strong correlation with experimental measurements (R^2^ = 0.96, NRMSE = 10.2%). The simulation underestimates the temperature accumulation in early layers (mean bias −12.8 °C below T_g_) due to the difference between surface-averaged infrared measurements and discrete nodal temperatures, while it overestimates the temperature in the final layers (mean bias 9.2 °C above T_g_) due to the instantaneous element activation at the prescribed deposition temperature.

## 4. Conclusions

This study successfully investigated the thermal deformation mechanisms in non-planar large-scale additive manufacturing of ABS. The integration of experimental measurements and finite element modeling led to the following key conclusions:

- Time-dependent cooling plays a dominant role in deformation behavior. Maintaining the deposited material above its glass transition temperature during printing significantly reduces both overall warping and skew deflection.
- Surface slope strongly influences deformation direction. The deflection tendency on negative-sloped surfaces were toward contraction. The exception at corner D was that folding occurred due to the time-dependent cooling. The observed deformation is primarily driven by the significant transient temperature gradients that exist between the initially deposited beads and the material currently being extruded at the nozzle. Therefore, it might be caused by antipodean-angled anisotropic folding. The positive-slope surface tends to the upward direction.
- The finite element method predictions demonstrate strong agreement with experimental measurements, with normalized root mean square errors (NRMSE) of approximately 11% for thermal deformation and 10% for temperature history.
- The deposited molten material and its temperature heated up the previous layer. It positively assisted in maintaining the layer temperature above T_g_ for a longer period.
- The surface that has a higher slope (25°) was seen with material sweeping due to the nozzle orientation. A solution could be suggested by adding a movement capability and control with a position control actuator, keeping the nozzle axis normal to the printing surface.

## Figures and Tables

**Table 1:** The mechanical properties of ABS [40].

Properties	Symbol	Unit	Value
Density	ρ	kg/m^3^	1060
Thermal Conductivity	k	W/m·K	0.177
Specific Heat	c	J/kg·K	2080
Emissivity	-	ε	0.87
Glass Transition Temperature	T_g_	°C	105
Coefficient of Thermal Expansion	α	°C^−1^	100 × 10^−6^

**Table 2:** Non-planar slicing parameter.

Parameter	Unit	Value
Nozzle diameter	mm	6
Deposition temperature	°C	240
Layer height	mm	4
Printing speed	mm/min	400
Overlap	mm	4
Adaptive slicing	-	Activated
Adaptive quality	%	75
For Non-planar Layers		
Maximum non-planar angle	degree	26
Maximum non-planar collision angle	degree	26
Minimum non-planar area	mm^2^	80
Maximum non-planar collision height	mm	18
Ignore collision size	mm^2^	5

**Table 3:** Comparison of the proposed finite element model with state-of-the-art models in terms of modeling approach, accuracy, and computational efficiency.

Study	FEM Software	Accuracy (FEM vs. Experimental)	Reference
Proposed Model	MSC Marc/Mentat 2021.4	11% error in deviation, 10.2% in temperature.	This study
Trofimov et al. (2022)	Abaqus	Thermal accuracy 1 °C; warpage average relative difference of ~2% to ~3.2% compared to the finest mesh.	[41]
Cattenone et al. (2018)	Abaqus	Average discrepancy of 12% between predicted and measured distortion.	[42]
Al Rashid & Koç (2023)	Digimat	High-dimensional accuracy, with errors % ranging from 0.13% to 0.76% for various dimensions.	[43]
Syrlybayev et al. (2021)	ANSYS	Error % for single material ranged from 2.5% to 60.9%, depending on the run. Multi-material error ranging from 1.4% to 9.5%.	[44]

## Data Availability

The original contributions presented in this study are included in the article. Further inquiries can be directed to the corresponding author.
